# Complete Genome Sequence of Ralstonia solanacearum Strain Bs715, a Member of Biovar 4 and a Strong Pathogen of Bacterial Wilt on Solanum lycopersicum

**DOI:** 10.1128/mra.00883-22

**Published:** 2023-01-23

**Authors:** HwangWeon Jeong, Hong-Il Ahn, Sung-Mi Kim, Young-Joo Seol, Hye-Jin Yoon, Jinjeong Lee, Su Jung Ra, Eunhee Kim, Myeong Jin Kang, Il-Pyung Ahn

**Affiliations:** a National Institute of Agricultural Sciences, Jeonju, South Korea; University of Arizona

## Abstract

Ralstonia solanacearum is a notorious pathogen of bacterial wilt on Solanum lycopersicum. Most isolates from diseased tomato tissues are biovar 3, and their genomes are publicly available; however, information on biovar 4 strains is limited. Here, the complete genome sequence of R. solanacearum Bs715, a biovar 4 strain, is presented.

## ANNOUNCEMENT

Ralstonia solanacearum is a causal agent of bacterial wilt and infects more than 200 plant species (1). Direct yield losses are frequently assessed at up to 90% for solanaceous crops, especially Solanum lycopersicum (2). Intensive investigations of molecular networks orchestrating plant-pathogen interactions (3–5) and extensive phylogenetic diversity analyzed by comparative analysis of bacterial genomes (6–9) have led to the concept of the R. solanacearum species complex (RSSC) encompassing three species and subspecies groups, including biovars (10). The biovar has been a traditional criterion determined by carbohydrate utilization (11, 12). Although the relationship between biovar and pathogenicity is hard to define, strains of biovar 3 are generally more virulent than those of biovar 4 (13) and are dominant in temperate regions (14). Our Bs715 strain is a member of biovar 4, phylotype Ι, race 1, and also is highly virulent on tomato plants. Sixty-four genomes of R. solanacearum strains are publicly accessible, but genome information on biovar 4 strains is limited.

R. solanacearum strain Bs715, which was isolated from symptomatic tomato vessel tissues collected in South Korea, was obtained from the Korean Agricultural Culture Collection, and its biovar was determined (12). A single colony of Bs715 was spread on casamino acid-peptone-glucose agar and grown at 28°C, and the cells were retrieved for DNA preparation (Nanobind CBB kit; Pacific Biosciences [PacBio], Menlo Park, CA, USA). DNA quality was satisfactory (Femto Pulse; Agilent, Santa Clara, CA, USA), and a Sequel library was constructed according to the 15-kbp template library preparation workflow (SMRTbell library preparation kit v3.0; PacBio). This library was analyzed on a Sequel sequencing system (PacBio). The total number of subreads analyzed was 162,139, and the number of bases was 2,391,967,749 (*N*_50_, 16,912 bp). The subreads were assembled into two circular contigs using the Microbial Assembly application in the PacBio single-molecule real-time (SMRT) Link program. A TruSeq library was prepared (TruSeq Nano DNA high-throughput library preparation kit; Illumina, San Diego, CA, USA) from the DNA described above and sequenced on a HiSeq X Ten system (Illumina). Reading depths for contig 1 and contig 2 were 173.2× and 260.7×, respectively. Pilon v1.21 was employed with default parameters to remove possible errors in our contigs based on the TruSeq reads; however, there was no flaw. Coding DNA sequences (CDSs) were annotated using the NCBI Prokaryotic Genome Annotation Pipeline (PGAP) (15).

The complete genome of Bs715 is composed of a 3,970,890-bp chromosome and a 2,064,370-bp megaplasmid, with GC contents of 66.6% and 67.0%, respectively. The annotation predicted 3,611 CDSs, 59 tRNAs, and 9 rRNAs on the chromosome and 1,605 CDSs, 7 tRNAs, and 3 rRNAs on the megaplasmid. Among a total of 5,216 CDSs, 190 genes encode components of the Bs715 protein secretion system, including the type 1 secretion system (T1SS) (32 genes), T2SS (27 genes), T3SS (15 genes), T4SS (28 genes), T5SS (15 genes), T6SS (38 genes), and flagella and pili (17 and 18 genes, respectively) (16). The megaplasmid contains most of the T3SS genes (14 genes) and flagellum genes (15 genes). The diverse repertoire of type III effectors (T3Es) anchored on the chromosome (21 genes) and the megaplasmid (36 genes) also suggests a broad host range of Bs715 (17). Bs715 contains 5 intact T3Es on the megaplasmid; 4 are pseudogenes, and the remaining 1 is absent in strain GMI1000.

### Data availability.

The genome information for the Bs715 chromosome and megaplasmid was deposited in GenBank with accession numbers CP102181 and CP102180, respectively. Raw TruSeq and Sequel reads were submitted to the NCBI SRA with accession numbers SRR20115436 to SRR20115439, under BioProject accession number PRJNA863051 and BioSample accession number SAMN29673691.
